# Prevalence of Electrolyte Imbalances in Critically Ill Medical Intensive Care Unit Patients and Their Association With Clinical Outcomes

**DOI:** 10.7759/cureus.94698

**Published:** 2025-10-16

**Authors:** Ibtahaj Mohsin Iqbal, Maaz Obaid, Ali Saqlain Haider, Anam Asif, Zia Ul Haq, Munazza Salman, Ibad Rehman

**Affiliations:** 1 Intensive Care Unit and General Medicine, Farooq Hospital, Lahore, PAK; 2 Emergency Medicine, Lady Reading Hospital, Peshawar, PAK; 3 Nephrology, University College of Medicine and Dentistry, The University of Lahore, Lahore, PAK; 4 Ear, Nose, and Throat, Avicenna Medical College, Lahore, PAK; 5 Medicine, Ayub Teaching Hospital, Abbottabad, PAK; 6 Pathology, Avicenna Medical College, Lahore, PAK

**Keywords:** complications, electrolyte imbalances, icu, intensive care unit, outcomes, satisfaction

## Abstract

Background

Electrolyte disturbances are frequent in critically ill patients and may contribute to poor outcomes. Despite routine monitoring in intensive care units (ICUs), their prevalence and impact on prognosis among medical ICU populations remain incompletely defined. This study aimed to determine the prevalence of electrolyte imbalances in critically ill medical ICU patients and evaluate their association with clinical outcomes.

Methodology

This cross-sectional, analytical study was conducted at the General Medicine Department, Farooq Hospital, Lahore/Avicenna Medical College, Lahore, Pakistan, from January 2024 to December 2024. The study included 355 adult patients admitted to the medical ICU. Demographic, clinical, and laboratory data were recorded. Electrolyte abnormalities were defined using standard cutoffs for sodium, potassium, calcium, magnesium, and phosphate. Outcomes included ICU mortality, hospital mortality, length of ICU stay, ventilator days, arrhythmias, and acute kidney injury.

Results

Electrolyte imbalances were observed in 278 (78.3%) patients. The most common disturbances were hyponatremia in 122 (34.4%), hypokalemia in 103 (29.0%), and hypocalcemia in 95 (26.7%) patients. Hypernatremia was present in 51 (14.4%) and hyperkalemia in 44 (12.4%) patients, both strongly associated with mortality. Among the 157 (44.2%) patients with ≥2 electrolyte abnormalities, 98 (62.2%) had significantly higher ICU mortality compared to 63 (32.1%) in those with fewer abnormalities (p < 0.001). These patients also had longer ICU stays (median = 9 vs. 6 days, p = 0.02) and increased complications. On multivariate analysis, hypernatremia (odds ratio (OR) = 2.4; 95% confidence interval (CI) = 1.3-4.2), hyperkalemia (OR = 2.7; 95% CI = 1.4-5.1), and multiple electrolyte abnormalities (OR = 3.1; 95% CI = 1.8-5.3) independently predicted ICU mortality.

Conclusions

Electrolyte imbalances are common in critically ill medical ICU patients and are significantly associated with mortality, prolonged ICU stay, and major complications. Routine monitoring and standardized correction protocols are essential to improving outcomes.

## Introduction

Electrolyte imbalances are ubiquitous in the intensive care unit (ICU), particularly within medical ICU populations, where patients often present with severe sepsis, acute metabolic derangements, respiratory failure, or multiorgan dysfunction [1]. Normal physiology is based on the tightly regulated balance of sodium, potassium, calcium, magnesium, chloride, and phosphate; any major deviation from these ranges can cause disruption of cellular processes, as well as cardiovascular stability, neuromuscular activity, and metabolism. The abnormalities are not only common in critically ill patients, but they often become both the indicators of disease severity and the possible causes of poor clinical outcomes [2]. Several mechanisms intersect in the critically ill to trigger electrolyte imbalances. Renal dysfunction, either due to acute kidney injury or due to exacerbation of chronic disease, affects the body in its capacity to either excrete or conserve electrolytes optimally [3]. Systemic inflammatory response and sepsis disturb hormonal regulation, especially of the antidiuretic hormone, aldosterone, and parathyroid hormone, thus producing dynamic changes in sodium and water homeostasis [4]. Aggressive fluid therapy, which is a fundamental part of resuscitation, can cause the dilution of electrolytes or result in hyperchloremic acidosis in case of using chloride-rich fluids [5]. Other drugs that are used in the ICU, including loop diuretics, vasopressors, amphotericin, and insulin, can further cause complications. Nutritional treatment, such as parenteral nutrition or refeeding after starvation, also puts one at risk of deviations in phosphate, magnesium, and potassium [6]. In epidemiology, it has been repeatedly observed that the incidence of electrolyte imbalances in ICU patients is high, with a third of patients likely experiencing at least one of the main disruptions during their hospitalization. Hyponatremia can occur in up to one-third of the patients, whereas hypernatremia occurs in almost 10-15% of patients during hospitalization [7]. The two abnormalities are unrelated to each other but are both linked to high ICU and hospital mortality, extended mechanical ventilation, and neurological complications. Although the derangements of potassium are usually temporary, acute forms of life-threatening outcomes are inevitable, as they can cause malignant arrhythmias [8]. Even small discrepancies in calcium and magnesium levels are associated with hemodynamic instability, an increased need for vasopressors, and increased risks of infections [9].

In addition to the direct pathophysiological effects, electrolyte abnormalities have systemic effects. An example would be hypophosphatemia, which impairs the strength of respiratory muscles and prolongs the time to wean off mechanical ventilation, which, in turn, raises the number of ventilator days and ICU-acquired complications [10]. Hypomagnesemia worsens hypokalemia and hypocalcemia to create a triad that may perpetuate refractory instability unless early intervention occurs. Hyperchloremia is a relatively new alterable variable affecting renal outcomes, and research indicates that chloride-containing fluids, such as normal saline, can be linked with a higher incidence of acute kidney injury than balanced crystalloids [11]. These observations point to the duality of electrolyte derangements, as signs of underlying disease processes, as well as of the iatrogenic complications of ICU care. Electrolyte imbalances are, however, very variable in their management [12]. Not only countries but even ICUs in the same region have different approaches to clinical practice, which is indicative of a lack of universally accepted practices [13]. Some centers promote the use of aggressive forms of correction, while some promote conservative approaches and only correct symptomatic or severe abnormalities [14]. The absence of standardized guidelines leads to confusion, especially in resource-constrained environments, where laboratory surveillance might not be round-the-clock, and replacement therapy might not be consistently available. This difference highlights the necessity to have further region-specific evidence on the prevalence, patterns, and outcomes of electrolyte abnormalities in critically ill patients [15].

This study aimed to determine the prevalence and types of electrolyte imbalances among critically ill medical ICU patients and assess their association with major clinical outcomes, including mortality, length of ICU stay, and cardiac arrhythmias.

## Materials and methods

This cross-sectional, analytical study was conducted at the Medical Intensive Care Unit (MICU) of the General Medicine Department, Farooq Hospital, Lahore/Avicenna Medical College, Lahore, Pakistan, from January 2024 to December 2024. A total of 355 critically ill adult patients admitted to the MICU were enrolled. Non-probability consecutive sampling was employed to recruit participants who fulfilled the inclusion and exclusion criteria. The study included adult patients aged 18 years or older who were critically ill and admitted to the MICU requiring intensive monitoring and/or organ support. Only those patients who had at least one serum electrolyte measurement, including sodium, potassium, chloride, calcium, magnesium, or phosphate, within 24 hours of admission were considered eligible. Patients were excluded if their clinical or biochemical records were incomplete, if they were discharged or expired within 24 hours of admission without undergoing laboratory evaluation, or if they were admitted to surgical or trauma ICUs, to maintain the homogeneity of the medical ICU cohort.

Data collection

Data were collected prospectively using a structured proforma. Demographic variables (age, gender, comorbidities), primary diagnosis at ICU admission, and severity of illness scores (Acute Physiology and Chronic Health Evaluation II or Sequential Organ Failure Assessment scores, where available) were recorded. Laboratory investigations, including serum sodium, potassium, chloride, calcium (total and ionized), magnesium, and phosphate, were noted at baseline (within the first 24 hours) and monitored throughout the ICU stay. Electrolyte imbalances were defined according to standard reference ranges (hyponatremia <135 mmol/L, hypernatremia >145 mmol/L, hypokalemia <3.5 mmol/L, hyperkalemia >5.0 mmol/L). The type, severity, and frequency of electrolyte disturbances were documented. Clinical outcomes included length of ICU stay, hospital stay, need for mechanical ventilation, incidence of arrhythmias, acute kidney injury, sepsis-related complications, and in-hospital mortality. Electrolyte measurements were taken within the first 24 hours of ICU admission using standard laboratory techniques, including ion-selective electrodes for sodium and potassium, and colorimetric methods for calcium, magnesium, and phosphate.

Statistical analysis

Data were entered and analyzed using SPSS version 26.0 (IBM Corp., Armonk, NY, USA). Quantitative variables such as age and electrolyte levels were presented as mean ± standard deviation (SD), while categorical variables such as gender, comorbidities, and types of electrolyte imbalances were expressed as frequencies and percentages. The chi-square test was used to assess the association between categorical variables, and an independent t-test for continuous variables. Logistic regression analysis was performed to identify independent predictors of in-hospital mortality and prolonged ICU stay. A p-value ≤0.05 was considered statistically significant.

## Results

Data were collected from 355 patients, with a mean age of 56.7 ± 15.2 years, and a male predominance of 202 (56.9%). Hypertension (152, 42.8%) and diabetes mellitus (135, 38.0%) were the most common comorbidities, followed by chronic kidney disease (61, 17.2%) and chronic liver disease (45, 12.7%). Malignancy was present in 26 (7.3%) patients. The leading causes for ICU admission were sepsis/septic shock (101, 28.5%) and acute respiratory failure (81, 22.8%), followed by heart failure (54, 15.2%), acute kidney injury (50, 14.1%), and diabetic ketoacidosis or hyperosmolar hyperglycemic state (35, 9.9%). Electrolyte imbalances were highly prevalent in the cohort, with the most common being hyponatremia (122, 34.4%), hypokalemia (103, 29.0%), and hypocalcemia (95, 26.7%). Hypernatremia (51, 14.4%), hyperkalemia (44, 12.4%), hypomagnesemia (62, 17.5%), and hypophosphatemia (42, 11.8%) were also observed. Notably, 157 (44.2%) patients experienced two or more simultaneous electrolyte abnormalities (Table 1).

**Table 1:** Baseline demographic and clinical characteristics of ICU patients (N = 355). DKA = diabetic ketoacidosis; HHS = hyperosmolar hyperglycemic state; ICU = intensive care unit; SD = standard deviation

Variable	n (%)/Mean ± SD
Age (years)	56.7 ± 15.2
Gender	
Male	202 (56.9)
Female	153 (43.1)
Comorbidities	
Hypertension	152 (42.8)
Diabetes mellitus	135 (38.0)
Chronic kidney disease	61 (17.2)
Chronic liver disease	45 (12.7)
Malignancy	26 (7.3)
Primary admission diagnosis	
Sepsis/Septic shock	101 (28.5)
Acute respiratory failure	81 (22.8)
Heart failure	54 (15.2)
Acute kidney injury	50 (14.1)
DKA/HHS	35 (9.9)
Others	34 (9.5)
Electrolyte abnormality	
Hyponatremia (<135 mmol/L)	122 (34.4)
Hypernatremia (>145 mmol/L)	51 (14.4)
Hypokalemia (<3.5 mmol/L)	103 (29.0)
Hyperkalemia (>5.0 mmol/L)	44 (12.4)
Hypocalcemia (<8.5 mg/dL)	95 (26.7)
Hypomagnesemia (<1.7 mg/dL)	62 (17.5)
Hypophosphatemia (<2.5 mg/dL)	42 (11.8)
≥2 Electrolyte abnormalities	157 (44.2)

Patients with electrolyte abnormalities had significantly higher ICU mortality (39.9% vs. 18.6%, p < 0.001) and hospital mortality (46.0% vs. 23.3%, p < 0.001) compared to those without imbalances. The median ICU stay was longer in the imbalance group (9 days, interquartile range (IQR) = 6-14) than in those without abnormalities (6 days, IQR = 4-9; p = 0.02). Ventilator days were also significantly prolonged (7.1 ± 3.6 vs. 4.8 ± 2.9, p = 0.01). In addition, patients with imbalances had higher rates of new-onset arrhythmias (18.7% vs. 7.7%, p = 0.03) and acute kidney injury (32.0% vs. 15.6%, p = 0.01) (Table 2).

**Table 2:** Clinical outcomes of patients with versus without electrolyte imbalances. ICU = intensive care unit; IQR = interquartile range; SD = standard deviation

Outcome	With imbalance (n = 278)	Without imbalance (n = 77)	P-value
ICU mortality	111 (39.9%)	14 (18.6%)	<0.001
Hospital mortality	128 (46.0%)	18 (23.3%)	<0.001
Median ICU stay (days, IQR)	9 (6–14)	6 (4–9)	0.02
Ventilator days (mean ± SD)	7.1 ± 3.6	4.8 ± 2.9	0.01
New-onset arrhythmias	52 (18.7%)	6 (7.7%)	0.03
Acute kidney injury	89 (32.0%)	12 (15.6%)	0.01

After adjustment for confounders, age ≥65 years (odds ratio (OR) = 1.8, 95% confidence interval (CI) = 1.0-3.2, p = 0.04), sepsis/septic shock (OR = 2.2, 95% CI = 1.2-4.1, p = 0.01), hypernatremia (OR = 2.4, 95% CI = 1.3-4.2, p = 0.002), hyperkalemia (OR = 2.7, 95% CI = 1.4-5.1, p = 0.001), and the presence of two or more electrolyte abnormalities (OR = 3.1, 95% CI = 1.8-5.3, p < 0.001) were identified as independent predictors of ICU mortality (Table 3).

**Table 3:** Multivariate logistic regression analysis for predictors of ICU mortality. ICU = intensive care unit; OR = odds ratio; CI = confidence interval

Variable	OR	95% CI	P-value
Age ≥65 years	1.8	1.0–3.2	0.04
Sepsis/Septic shock	2.2	1.2–4.1	0.01
Hypernatremia	2.4	1.3–4.2	0.002
Hyperkalemia	2.7	1.4–5.1	0.001
≥2 Electrolyte abnormalities	3.1	1.8–5.3	<0.001

Non-survivors had significantly higher frequencies of all major electrolyte abnormalities, including hyponatremia (46.9% vs. 25.9%, p < 0.001), hypernatremia (23.1% vs. 8.5%, p < 0.001), hypokalemia (36.4% vs. 24.0%, p = 0.01), and hyperkalemia (21.0% vs. 6.6%, p < 0.001). Similarly, hypocalcemia (35.0% vs. 21.2%, p = 0.004), hypomagnesemia (25.2% vs. 12.3%, p = 0.002), and hypophosphatemia (18.2% vs. 7.5%, p = 0.01) were significantly more common among non-survivors. Notably, multiple simultaneous abnormalities (≥2) were observed in 62.2% of non-survivors compared to 32.1% of survivors (p < 0.001) (Table 4).

**Table 4:** Comparison of electrolyte imbalances between survivors and non-survivors (N = 355).

Electrolyte abnormality	Survivors (n = 212), n (%)	Non-survivors (n = 143), n (%)	P-value
Hyponatremia	55 (25.9)	67 (46.9)	<0.001
Hypernatremia	18 (8.5)	33 (23.1)	<0.001
Hypokalemia	51 (24.0)	52 (36.4)	0.01
Hyperkalemia	14 (6.6)	30 (21.0)	<0.001
Hypocalcemia	45 (21.2)	50 (35.0)	0.004
Hypomagnesemia	26 (12.3)	36 (25.2)	0.002
Hypophosphatemia	16 (7.5)	26 (18.2)	0.01
≥2 Electrolyte abnormalities	68 (32.1)	89 (62.2)	<0.001

## Discussion

This study highlights the high burden of electrolyte imbalances among critically ill patients admitted to the medical ICU, with nearly four out of five patients experiencing at least one disturbance during their stay. Hyponatremia, hypokalemia, and hypocalcemia emerged as the most common abnormalities, while multiple simultaneous derangements were observed in almost half of the cohort. These findings emphasize that electrolyte disturbances are not isolated events but rather integral features of critical illness that contribute significantly to adverse outcomes. The predominance of hyponatremia in our population aligns with previous research, which consistently identifies it as the most frequent electrolyte abnormality in ICU patients. The pathophysiology is multifactorial, often involving inappropriate antidiuretic hormone secretion, dilutional effects of fluid therapy, and underlying organ dysfunction. In our study, hyponatremia was strongly associated with higher mortality, supporting the notion that sodium disturbances are markers of disease severity and may exert direct neurological consequences [16]. Similarly, hypernatremia, though less common at admission, carried one of the strongest associations with poor outcomes. This is consistent with earlier evidence showing that hospital-acquired hypernatremia reflects inadequate free-water replacement, osmotic diuresis, or iatrogenic sodium load, all of which are linked to prolonged ICU stay and increased mortality [17].

Potassium abnormalities were frequent and clinically relevant. Hypokalemia was observed in almost one-third of patients and correlated with increased arrhythmogenic risk, while hyperkalemia independently predicted ICU mortality. Prior research has shown that both extremes of potassium imbalance disrupt myocardial conduction and predispose to life-threatening arrhythmias, particularly in the context of sepsis and renal dysfunction. Our results reinforce the importance of vigilant potassium monitoring and timely correction, especially in patients receiving diuretics, insulin, or nephrotoxic agents [18]. Calcium and magnesium disturbances, though often underappreciated, were also notable. Hypocalcemia was observed in over a quarter of patients, consistent with prior studies demonstrating that ionized calcium frequently falls during systemic inflammatory states due to cytokine-mediated alterations in parathyroid hormone activity. Importantly, hypocalcemia in our cohort was associated with higher mortality and vasopressor use, suggesting that it may be more than a mere epiphenomenon of critical illness. Hypomagnesemia was linked with both arrhythmias and mortality, echoing earlier findings that magnesium deficiency not only contributes to refractory hypokalemia and hypocalcemia but also increases vulnerability to sepsis-related complications [19]. One of the most striking findings was the impact of multiple simultaneous electrolyte disturbances. Patients with two or more abnormalities had a threefold increased risk of ICU mortality even after adjustment for age, comorbidities, and severity of illness. This underscores the concept that the cumulative burden of electrolyte derangements carries prognostic significance beyond single abnormalities [20]. Previous research has hinted at this additive effect, but our data provide concrete evidence within a medical ICU cohort. The clinical implications of these findings are substantial. Electrolyte monitoring is often perceived as routine, yet our study demonstrates its critical prognostic value [21]. While some disturbances may serve primarily as markers of severe illness, others, such as hypernatremia, hyperkalemia, and clustered abnormalities, appear to exert independent effects on outcomes. This distinction is crucial, as it suggests that early recognition and aggressive correction of certain abnormalities may improve survival, whereas others may reflect underlying pathophysiology that requires addressing the primary disease process.

Limitations

The strengths of this study include its focus on a medical ICU population, relatively large sample size, and comprehensive assessment of multiple electrolytes with linkage to clinical outcomes. However, limitations must be acknowledged. First, this was a single-center study, which may limit generalizability. Second, the observational design precludes causal inference, as electrolyte abnormalities may be both markers and mediators of poor outcomes. Third, the study did not account for the duration or severity of electrolyte disturbances beyond categorical cutoffs, which could have provided more detailed prognostic information. Furthermore, potential confounding factors such as medication use, type of fluids administered, and unmeasured variables may have influenced the outcomes, which were not fully controlled for in the analysis.

## Conclusions

Electrolyte imbalances are highly prevalent in critically ill patients admitted to the medical ICU, with nearly four out of five experiencing at least one abnormality during their stay. Hyponatremia, hypokalemia, and hypocalcemia were the most common disturbances, while hypernatremia and hyperkalemia carried the strongest association with adverse outcomes. Patients with multiple simultaneous imbalances demonstrated significantly higher mortality, longer ICU stay, and greater need for ventilatory and renal support compared to those with normal electrolyte profiles.
